# Protocol for a hybrid type 2 cluster randomized trial of trauma-focused cognitive behavioral therapy and a pragmatic individual-level implementation strategy

**DOI:** 10.1186/s13012-020-01064-1

**Published:** 2021-01-07

**Authors:** Aaron R. Lyon, Michael D. Pullmann, Shannon Dorsey, Carol Levin, Larissa M. Gaias, Stephanie K. Brewer, Madeline Larson, Catherine M. Corbin, Chayna Davis, Ian Muse, Mahima Joshi, Rosemary Reyes, Nathaniel J. Jungbluth, Rachel Barrett, David Hong, Michael D. Gomez, Clayton R. Cook

**Affiliations:** 1 University of Washington, 6200 NE 74th Street, Suite 100, Seattle, WA 98115 USA; 2 Department of Psychology, University of Massachusetts, Lowell, 850 Broadway St, Lowell, MA 01854 USA; 3 University of Minnesota, 250 Education Sciences Building, 56 East River Road, Minneapolis, MN 55455 USA; 4 Seattle Children’s Hospital, 4800 Sand Point Way NE, Seattle, WA 98105 USA; 5 Washburn Training Institute, 1100 Glenwood Ave, Minneapolis, MN 55405 USA; 6 Psychological Sciences, Texas Tech University, 2700 18th Street, Lubbock, TX 79410 USA

**Keywords:** Individual determinants, Implementation strategy, Theory of planned behavior, Health action process approach, Education sector, Mental health

## Abstract

**Background:**

More than two-thirds of youth experience trauma during childhood, and up to 1 in 5 of these youth develops posttraumatic stress symptoms that significantly impair their functioning. Although trauma-focused cognitive behavior therapy (TF-CBT) has a strong evidence base, it is rarely adopted, delivered with adequate fidelity, or evaluated in the most common setting where youth access mental health services—schools. Given that individual behavior change is ultimately required for successful implementation, even when organizational factors are firmly in place, focusing on individual-level processes represents a potentially parsimonious approach. Beliefs and Attitudes for Successful Implementation in Schools (BASIS) is a pragmatic, motivationally focused multifaceted strategy that augments training and consultation and is designed to target precise mechanisms of behavior change to produce enhanced implementation and youth clinical outcomes. This study protocol describes a hybrid type 2 effectiveness-implementation trial designed to concurrently evaluate the main effects, mediators, and moderators of both the BASIS implementation strategy on implementation outcomes and TF-CBT on youth mental health outcomes.

**Methods:**

Using a cluster randomized controlled design, this trial will assign school-based mental health (SMH) clinicians and schools to one of three study arms: (a) enhanced treatment-as-usual (TAU), (b) attention control plus TF-CBT, or (c) BASIS+TF-CBT. With a proposed sample of 120 SMH clinicians who will each recruit 4–6 youth with a history of trauma (480 children), this project will gather data across 12 different time points to address two project aims. Aim 1 will evaluate, relative to an enhanced TAU condition, the effects of TF-CBT on identified mechanisms of change, youth mental health outcomes, and intervention costs and cost-effectiveness. Aim 2 will compare the effects of BASIS against an attention control plus TF-CBT condition on theoretical mechanisms of clinician behavior change and implementation outcomes, as well as examine costs and cost-effectiveness.

**Discussion:**

This study will generate critical knowledge about the effectiveness and cost-effectiveness of BASIS—a pragmatic, theory-driven, and generalizable implementation strategy designed to enhance motivation—to increase the yield of evidence-based practice training and consultation, as well as the effectiveness of TF-CBT in a novel service setting.

**Trial registration:**

ClinicalTrials.gov registration number NCT04451161. Registered on June 30, 2020.

**Supplementary Information:**

The online version contains supplementary material available at 10.1186/s13012-020-01064-1.

Contributions to the literature

- Research has demonstrated that even when organizational factors are firmly in place, individual behavior change is critical to implementation. However, few implementation strategies have been designed and tested surrounding their impact on precise, theoretically derived mechanisms of individual behavior change.
- This study will be the first to test a pragmatic, theory-driven, and generalizable implementation strategy in the context of improving clinician use of an evidence-based trauma intervention in the education sector, the most common setting for youth mental health service delivery.
- Findings will contribute to gaps in the implementation literature surrounding the utility of pragmatic implementation strategies, the mechanisms through which strategies operate, and the effectiveness of trauma interventions in accessible service settings.

## Background

### Childhood trauma exposure and treatment

More than two-thirds of youth experience trauma during childhood, with one-third experiencing multiple traumatic events [1]. Up to 1 in 5 trauma-exposed youth develops posttraumatic stress symptoms or posttraumatic stress disorder (PTSD) that results in significant impairment in daily functioning [1]. Most youth with trauma-related mental health disorders do not receive treatment, and when they do access care, the services they receive are not evidence-based or delivered with adequate fidelity [2, 3].

Trauma-focused cognitive-behavioral therapy (TF-CBT) has the strongest evidence base of any child trauma treatment, with over 16 randomized trials demonstrating a range of positive outcomes across sex, age range, and ethnic and cultural groups for reduced symptoms of PTSD, anxiety, depression, and trauma-related behavioral problems [4, 5]. Evidence exists for sustained impact 1 year later [6]. Despite its efficacy, there are no effectiveness studies evaluating TF-CBT in schools. This is concerning, as there are attempts to scale-up TF-CBT in schools and other community settings through a variety of national initiatives [7, 8], even in the absence of evidence for effects in the education sector.

### School mental health and the implementation gap

Efficacious mental health services such as TF-CBT are unlikely to yield public health impact unless they are consistently implemented in accessible settings. School-based mental health (SMH) services account for 50–80% of all youth mental health services in the USA [9–11]. Despite this, treatments found to be efficacious in other settings (e.g., community mental health) have not been “scaled-out” to the educational sector [12]. Like other service sectors, SMH service delivery is characterized by uneven adoption and insufficient fidelity to evidence-based services [13–16]. As a consequence, the potential of EBTs, like TF-CBT, to reduce symptoms, improve functioning, and ultimately impact public health is significantly curtailed.

### Multilevel implementation determinants

As outlined in the Exploration, Preparation, Implementation, Sustainment (EPIS) framework [17], implementation success depends on both individual- and system-level determinants (i.e., barriers and facilitators). Although the importance of organizational influences on implementation is well established [17, 18], organizational change is time consuming and expensive [19], requiring years of consistent and sustained effort to show effects. In contrast, focusing on individual-level processes represents a potentially parsimonious and pragmatic approach to improve the quality of trauma-focused services. Individual behavior change is ultimately required for successful EBT implementation, even when organizational factors are firmly in place [20–22]. Indeed, some studies have found that individual factors (especially attitudes) may be significantly more predictive of the use of EBT than organizational factors (e.g., organizational culture, implementation climate) [23, 24]. Although individual attitudes and behaviors are embedded within larger contexts [25], individual barriers may be more malleable and proximal to EBT implementation.

### Individual-level implementation strategies

Research on individual-level implementation strategies has largely focused on training and consultation, which are necessary but insufficient to ensure successful EBT implementation [18, 26]. Many providers fail to successfully deliver an EBT even after receiving high-quality training and consultation [27]. Clinicians often remain resistant or ambivalent to change [28, 29] or lack the sustained self-efficacy or motivation to overcome perceived barriers to implementation [28].

To influence provider behaviors in response to EBT training and consultation, the current project applies two well-established theories of behavior change: the Theory of Planned Behavior (TPB) and Health Action Process Approach (HAPA) [30–32], which include motivational and volitional phases of behavior change and have been applied to implementation efforts [33, 34]. We selected these behavior change theories because of their clear explication of individual-level factors impacting behavior and their strong empirical support [35–38]. The central tenet of TPB is that one of the best predictors of behavior is a person’s behavioral intentions [30, 32] or an individual’s motivation or conscious plan to exhibit a particular behavior. Behavioral intentions, in turn, are a function of an individual’s attitudes (cognitive appraisals of the behavior), subjective norms (social pressure to perform the behavior), and self-efficacy (an individual’s confidence about performing a behavior).

The ability to predict behavior using TPB significantly increases with the addition of volitional strategies that support individuals in enacting the behaviors they are motivated to exhibit [39]. HAPA articulates volitional strategies including action planning, or specifying the “when,” “where,” and “how” of a behavior, and problem-solving planning, or articulating how to overcome barriers that interfere with one’s action plan [40]. When applied in combination, these two volitional strategies increase maintenance self-efficacy (i.e., a person’s optimistic beliefs about their capability to overcome barriers that arise while attempting to enact and maintain behavior) and facilitate the link between intentions and behavior, thus increasing the likelihood that specific implementation outcomes (e.g., adoption, fidelity) will occur [41].

Even when TPB has been used to guide process evaluations, efforts have explored traditional implementation strategies such as clinician education and performance-based feedback [42–47]. Aspects of HAPA, such as planning interventions, have demonstrated success in single-case or correlational implementation studies but have not been evaluated in randomized trials. Considering the promise of TPB and HAPA for shifting behavior, these theories should inform the development and testing of novel individual-level implementation strategies.

### Beliefs and Attitudes for Successful Implementation in Schools

**Fig. 1:**
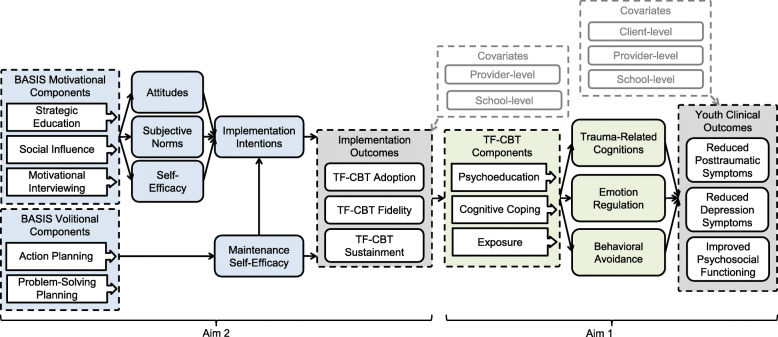
BASIS and TF-CBT intervention components, hypothesized mechanisms of change, and target outcomes. Mechanisms appear in boxes with rounded corners. Blue = BASIS. Green = TF-CBT. Gray = outcomes

Grounded in TPB and HAPA (see Fig. 1) [30, 31, 35], the Beliefs and Attitudes for Successful Implementation in Schools (BASIS) strategy serves to augment EBT training and consultation. BASIS aims to increase implementation intentions and outcomes by shifting clinician attitudes, subjective norms, and self-efficacy during the motivational phase and maintaining self-efficacy during the volitional phase of behavior change. BASIS is delivered immediately prior to and immediately after EBT training. In addition, clinicians receive an individualized BASIS booster roughly 15 days post-training that is tailored to whether implementation has been initiated. Within the EPIS framework, BASIS sits at the intersection of the preparation/adoption and active implementation phases [17].

Figure 1 displays core BASIS and TF-CBT components, as well as their respective mechanisms of change (components are described in the Approach). Evaluation of implementation mechanisms is critical to ensuring the most effective and streamlined implementation strategies. There are very few theoretically informed implementation strategies that target precise mechanisms of behavior change [48, 49]. BASIS mechanisms are organized according to whether they are motivational or volitional. Each of the motivational mechanisms drawn from TPB (i.e., attitudes, subjective norms, self-efficacy) represents a malleable individual determinant linked to increased intentions to participate in training and consultation. The volitional mechanism derived from HAPA (i.e., maintenance self-efficacy) captures a critical determinant of the likelihood of clinicians initiating and maintaining implementation following training [50]. Each of the motivational and volitional mechanisms informs a specific component of the BASIS implementation strategy, described below.

In previous pilot work, BASIS has demonstrated large effects on target mechanisms and overall feasibility [23]. For instance, when a preliminary version of BASIS was delivered to 1181 educators in 62 schools, pre-post surveys showed that BASIS led to more favorable post-intervention EBT attitudes (*d* = 1.03) [51]. Attitudes, in turn, were associated with two measures of EBT fidelity (*d* = .51; *d* = .67). In a small-scale randomized trial with SMH clinicians, 23 results indicated that BASIS was highly feasible, acceptable, and contextually appropriate. Further, moderate to large effects at post-training for BASIS mechanisms of change encouraged the current trial.

### Objectives and aims

The objective of this hybrid type 2 randomized effectiveness-implementation trial is to simultaneously examine the theoretical mechanisms through which a clinical intervention (TF-CBT) and an implementation strategy (BASIS) impact clinical and implementation outcomes, respectively [52].

#### Aim 1: experimentally evaluate the effectiveness and cost-effectiveness of TF-CBT in schools versus an enhanced treatment-as-usual condition

Aim 1 will evaluate, relative to control, the effects of TF-CBT conditions on TF-CBT’s identified mechanisms of change (trauma-related cognitions, emotion regulation, behavioral avoidance), differences in child mental health outcomes, and intervention costs and cost-effectiveness.

#### Aim 2: experimentally evaluate the impact and cost-effectiveness of BASIS versus attention control

Aim 2 will evaluate the main effects of BASIS, relative to control, on its theoretical implementation mechanisms, implementation outcomes, as well as costs and cost-effectiveness. We will also evaluate “hypothesis-defying residuals” (i.e., clinicians whose implementation behaviors are unaccounted for by the theoretical model) to further refine our BASIS theory of change.

## Method

**Fig. 2:**
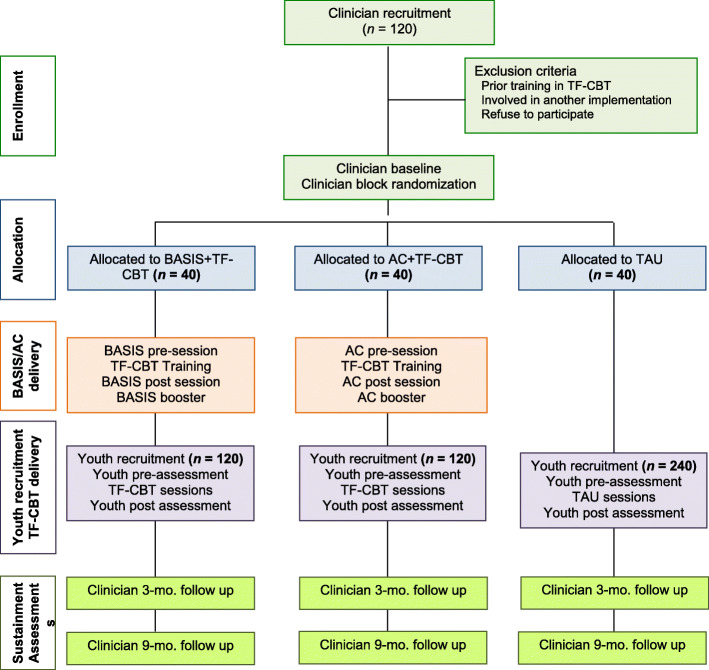
CONSORT diagram

In this hybrid type 2 effectiveness-implementation stratified cluster randomized trial, a single clinician from each participating school will be randomized to BASIS plus TF-CBT (BASIS+TF-CBT), attention control plus TF-CBT (AC + TF-CBT), or enhanced treatment as usual (TAU) (see CONSORT diagram in Fig. 2 and Additional Files 1 and 2 for completed CONSORT and SPIRIT checklists). Participants will be recruited over three waves, one per year. Youth participants will be assigned to condition based on their school clinician’s condition. Surveys, interviews, and direct observations will be used to evaluate the impacts and costs of each intervention. In addition, sequential mixed-methods data collection [53] will explore how mechanisms are linked to implementation outcomes for “hypothesis-defying residuals” (e.g., clinicians whose intentions to implement are inconsistent with their documented implementation behaviors).

Additional File 3 contains human subjects’ approval. All data collected will be de-identified and stored on secure servers accessible only to members of the research team. Any adverse events or protocol modifications will be tracked and, when indicated, reported in a timely manner to the institutional review board and sponsor.

### Changes due to COVID-19

As a result of the COVID-19 pandemic—and with approval from the study’s Program Officer—delivery of BASIS, AC, and training for clinicians in TF-CBT or enhanced TAU was switched from in-person to remote for the duration of the project. In addition, the study was adjusted to allow clinicians to provide virtual services to youth as indicated by their school district/employer policies.

### Participants and recruitment

#### Clinicians

Clinician participants will include 120 SMH providers (40 per wave). SMH providers will consist of school psychologists, social workers, mental health counselors, etc. recruited from elementary, middle, and high schools from economically and ethnically diverse districts. One clinician per school will be recruited with the assistance of district administrators. The research team will contact eligible participants to describe the purpose of the study, research procedures, and incentives. Clinicians will be included if they (a) serve in a professional role to provide school-based mental health services, (b) hold a graduate degree or equivalent certification or experience, (c) have not previously received formal training in TF-CBT, and (d) are not actively receiving support to implement another related intervention.

#### Students

Youth participants will include 480 students (160 per wave), recruited by TF-CBT and TAU therapists. Youth will meet TF-CBT eligibility criteria, including (a) be enrolled in grades 3–12 and (b) have traumatic event exposure (e.g., exposure to violence) and (c) significant post-traumatic stress symptoms. SMH clinicians will recruit students by screening through their standard referral pathways (e.g., teacher- or self-referral; screening) for PTSD symptoms. Once students are identified, clinicians will ask caregivers whether they are open to being contacted by the research team. For caregivers who agree, contact information will be relayed to the team, who will follow-up by phone to describe the research project, estimated time to participate, participant compensation, and obtain consent (see Additional File 4 for all consent forms). For caregivers who consent, research staff will explain the study to students, answer questions, and obtain assent for participation. Four to six students will be recruited by each clinician. Based on previous research [54, 55], it is expected that students will be ~ 50% female and representative of the schools in which they are enrolled.

### Randomization

Clinician random assignment will occur at the school level, with one clinician per school recruited. We use this strategy to minimize analytic difficulties associated with reliably partitioning school and clinician variance with small numbers of clinicians per school and eliminate the risk of condition contamination. Clinicians and TF-CBT trainers/consultants will be blind to their BASIS/AC condition assignment. To ensure comparability of conditions at baseline, minimize potential confounding, and maximize accuracy of effect estimates, we will use a stratified randomization design, carried out by the study’s lead methodologist (MP). We will collect baseline clinician data on implementation intentions and mechanisms (e.g., attitudes, norms, self-efficacy), clinician years of experience, and school characteristics (school size, attendance rates, free/reduced lunch rates, percentage White, disciplinary rates). We will use the nearest neighbor algorithm to generate paired distance estimates between each clinician and then select matched groups of three based on smallest distance estimates. Each group of three will be randomly assigned to each of the three conditions.

### Clinical interventions

Both BASIS and AC will bookend a TF-CBT virtual training and will be followed by TF-CBT consultation and online booster session.

#### TF-CBT

TF-CBT is a 12–16 session intervention for children aged 3 to 18 years with trauma-exposure and related mental health sequelae. TF-CBT includes individual sessions for the youth, individual sessions for parents, and conjoint sessions that include both the youth and parent. TF-CBT has established training and consultation protocols, as well as a psychometrically strong objective fidelity instrument (see Table 1) [56]. All clinicians assigned to one of the TF-CBT conditions will participate in “gold standard” virtual TF-CBT training including completing of an online, self-paced 8–10 h didactic training, a virtual live 3-day training (3–4 h per day) with a certified TF-CBT trainer, and 6 months of post-training consultation. Consultation groups (6–8 providers/each) will be formed within condition to avoid contamination.

**Table 1:** BASIS strategy components

**Motivational components (TPB mechanisms)**	
1. **Strategic education (attitudes)**	
a. Connecting EBP to student success	
b. Problems with implementing non-EBPs	
c. Addressing common myths about EBPs	
d. Evaluating evidence for practices	
e. Promoting understanding of fidelity for EBP	
2. **Social influence (subjective norms)**	
a. Providing normative information	
b. Testimonials from experts	
c. Testimonials from similar others	
d. Evoking public commitments	
3. **Motivational interviewing (self-efficacy)**	
a. Professional values clarification activity	
b. Pros and cons activity to elicit change talk	
c. Anticipating implementation barriers	
d. Values-directed goal setting	
e. “Ruler questions” (e.g., how confident are you?)	
**Volitional components (HAPA mechanism)**	
4. **Action planning and problem-solving planning (maintenance self-efficacy)**	
a. Action planning to initiate implementation	
b. Problem-solving planning to overcome barriers	

#### TAU

The enhanced TAU condition is intended to ensure safety and support SMH providers who have variable levels of experience treating trauma directly and reduce unwanted variance in TAU. The research team developed a brief protocol to provide guidelines for psychoeducation and post assessment connection and support as scaffolding for usual care in schools. To maintain internal validity, this companion protocol does not include in depth attention to the elements of TF-CBT (e.g., exposure) hypothesized to be responsible for its effects. TAU scaffolding will be provided via an online presentation with a live question and answer section at the conclusion.

### BASIS strategy

The BASIS multifaceted implementation strategy is group-based and interactive, with a pre-training session (~ 3 h) delivered prior to TF-CBT training, a post-training session (~ 90 min) delivered immediately after training, and an online booster ~ 15 days post-training. Below, we describe BASIS motivational and volitional components, as well as the BASIS structure and fidelity assessment. Table 1 displays all BASIS components.

#### BASIS motivational components

The first BASIS component involves *strategic education* focused on increasing beliefs and attitudes about the benefits of EBT and intervention fidelity to improve attitudes. Examples of popular but ineffective practices (e.g., learning styles) are used to help clinicians identify cognitive shortcuts that enhance vulnerability to adopting non-EBTs. In addition, participants are prompted to reflect on the importance of fidelity across a range of professions (e.g., engineering, farming, aviation).

Second, BASIS includes social influence techniques to alter perceptions of subjective norms. Evidence-based social influence strategies consist of two broad categories: (1) social proofing messages that use data or testimonials to describe the behavior or attitudes of others and (2) strategies to induce cognitive dissonance. Social proofs are most influential from individuals with whom they closely identify [57], especially when testimonials speak to the usefulness of the specific behavior [58, 59]. Strategies to induce cognitive dissonance operate on the premise that individuals strive for consistency between their attitudes and actions [60]. Thus, desired behaviors can be increased by evoking commitments that are active (vs. passive), public (vs. private), and voluntary (vs. coerced) [61, 62]. These strategies are integrated throughout BASIS. For instance, normative data and testimonials are used to normalize clinician experience of barriers to EBT implementation (e.g., lack of time, low administrative support), express commitment to problem solve barriers, and debunk common myths about EBTs.

Third, motivational interviewing (MI) is used to enhance self-efficacy. MI is a nondirective, patient-centered approach with strong evidence for building engagement and commitment for behavior change [63–68]. The BASIS facilitator utilizes group MI techniques by adopting an empathic, nondirective, and person-centered style to elicit self-motivational statements and encourage “change talk” (i.e., statements about making behavioral changes). Participants engage in a values affirmation activity [69, 70] that has been shown to decrease defensiveness toward change and enhance motivation [71]. To further enhance self-efficacy, participants also anticipate barriers that may arise in implementation and collaborate to generate solutions to those barriers and engage in decisional balance activities to reflect on the pros and cons of changing or not changing.

#### BASIS volitional components

To address the intention-behavior gap, BASIS includes volitional planning interventions derived from HAPA to increase the likelihood that clinicians will maintain self-efficacy and act upon their intentions by enacting implementation behaviors. Specifically, action planning and problem-solving planning have been shown to facilitate health behaviors such as breast cancer self-examinations, medication adherence, exercise, and healthy eating [72–77]. Action planning supports translation of intentions into actions through detailed planning of how to perform behaviors in specific contexts. Problem-solving generates solutions in response to both situational and internal (e.g., cognitive) barriers to facilitate follow through with the action plan. In combination, action planning and problem-solving planning increase the likelihood that implementation intentions translate into behavior change [39]. Action planning and problem-solving planning occur immediately post-EBT training.

#### BASIS structure

The BASIS *pre-training session* targets attitudes, subjective norms, and self-efficacy via the motivational components listed above. The pre-training opens with the facilitator engaging SMH providers in an activity to clarify their professional values (*MI component*). *Strategic education* components are not delivered didactically, but rather to facilitate interaction among participants. Open-ended questions are used to elicit change talk. Testimonials are interspersed throughout (*social influence*). At the end of the pre-training session, providers collaborate with each other to develop an individualized menu of potential solutions to common implementation barriers that they can select from when encountering challenges to adopting and delivering new practices with fidelity. Last, they set value-congruent goals related to participation and engagement in the upcoming EBT training.

The BASIS *post-training* session includes volitional strategies shown in prior research to maintain implementation intentions and facilitate actual enactment of behavior change. Specifically, clinicians are supported to develop action plans and problem-solving plans. Clinicians are provided with an action planning template to detail precisely what TF-CBT components, how, with whom, where/when, and the environmental cues and resources needed to initiate delivery of TF-CBT with fidelity. The problem-solving plan involves clinicians anticipating situational and internal barriers and generating solutions to overcome those barriers to develop personalized *if-then* plans that can be used when confronted with specific barriers.

The BASIS online booster is delivered at approximately 15 days post-training, a time point when clinicians’ implementation intentions and behaviors may first weaken [78]. The aim of the BASIS booster is to provide adaptive content to clinicians to either increase intentions to implement or maintain self-efficacy to implement the EBT, depending on whether or not clinicians have initiated TF-CBT implementation. For instance, clinicians who have not yet initiated implementation will (1) be provided with a distilled version of the pre-training BASIS content (improving attitudes, subjective norms, and self-efficacy) and (2) be supported to revise their action and problem-solving plans with specific attention to components of these plans that were not aligned with their implementation intentions or the constraints of their service settings.

#### BASIS fidelity

A BASIS fidelity tool [78] will be used to rate recordings of BASIS delivery by trained research assistants. Each recording will be coded independently by two different raters and disagreements resolved through consensus dialog to capture facilitator adherence and participant responsiveness via engagement [79, 80].

### Attention control

Providers randomly assigned to AC condition will receive a 3-h pre-training, 90-min post-training session, and an online booster roughly 15 days post-training to mirror the duration of BASIS and control for dose, information provided, and interventionist effects. The AC condition will be delivered by the same facilitator as BASIS to control for facilitator effects. Content will be didactic, as is typical in trainings for SMH clinicians [81, 82]. The AC pre-training session will provide content on the definition of EBT, how EBTs are established, why clinicians should use EBTs, clinical outcomes associated with different EBTs, and defining different dimensions of fidelity. The post-training session will involve having control clinicians reflect on TF-CBT and its core components and discuss the outcomes associated with TF-CBT. The AC booster will prompt clinicians to reflect on and describe TF-CBT and identify and define each of its core components.

### Clinician data collection

Clinician data collection will span the active implementation and sustainment phases (18 months in total). Data will include clinician quantitative surveys and qualitative interviews, fidelity assessments of recorded TF-CBT sessions (via objective coding), and ratings of TF-CBT case presentations completed by TF-CBT consultants. Data collection will be incentivized in both the implementation and sustainment phases.

#### Quantitative surveys

Clinician surveys will be administered via a secure web-based system at 12 time points for BASIS/AC and 9 time points for TAU, beginning in the fall of each year. Clinicians will self-report their demographic characteristics, BASIS mechanisms (attitudes, subjective norms, self-efficacy, maintenance self-efficacy), implementation intentions, organizational moderators (implementation climate, leadership), and TF-CBT sessions delivered. For BASIS/AC, surveys will be administered at baseline through post-training (T1 prior to the self-paced course, T2 after the self-paced course, T3 after BASIS/AC pre-training session, T4 after the TF-CBT virtual training, and T5 at end of the BASIS/AC post-training session), 2 months from baseline and after the BASIS/AC booster (T6), winter (T7), spring (T8), and end of school year (T9), as well as three sustainment time points during the subsequent year (T10 in fall or year 2, T11 in winter, and T12 in spring). For TAU, we will administer the T1, T5, and T6–T12 surveys.

#### TF-CBT fidelity assessments

To assess TF-CBT fidelity, clinicians will record all sessions for participating TF-CBT and TAU students. Three sessions (one from each phase of TF-CBT) will be randomly selected and coded for fidelity using an established, systematic TF-CBT coding protocol (Table 2)*.*

**Table 2:** Study measures by construct

Construct	Measure	Type	Informant	Timing
Demographics and context				
Provider demographics	Age, gender, race/ethnicity, education level, years of experience, etc.	Q	C	T0
School characteristics/context	School size, % eligible for free lunch, racial/ethnic composition, etc.	R	R	T0
Mediators (mechanisms) and moderators for BASIS and TF-CBT				
Attitudes towards EBT	Evidence-Based Attitudes Scale (EBPAS) [83]	Q	C	T1-T12
Subjective norms	The modified Subjective Norms measure [42, 84]	Q	C	T1-T12
Self-efficacy	Modified Teacher Self-Efficacy Scale [85]	Q	C	T1-T12
Maintenance self-efficacy	Re-administration of the Modified Teacher Self-Efficacy Scale [59]			
Intentions to implement EBT	The Modified-Intentions to Use Scale [86]	Q	C	T1-T12
Trauma-related cognitions	Child Post-Traumatic Cognitions Inventory (CPTCI) [87]	S	Y	ST1-ST3
Emotion regulation	Emotional Regulation Questionnaire (ERQ) [88]	S	Y	ST1-ST3
Behavioral avoidance	Posttraumatic Avoidance Behavior Questionnaire (PABQ) [89]	S	Y	ST1-ST3
Implementation climate	The Implementation Climate Scale (ICS) [90, 91]	Q	C	T6, T10
Implementation leadership	The Implementation Leadership Scale (ILS) [91, 92]	Q	C	T6, T10
Implementation determinants	Semi-structured interviews with clinicians	I	C	Between T5 and T6
Implementation and sustainment outcomes				
BASIS/AC fidelity	Videotaped coding of BASIS facilitation (adherence and competence)	O	O	T2
TF-CBT consultation participation	Consultation sessions attended, days post-training to dropout	R	N	T2-T4
TF-CBT adoption	Date of first TF-CBT session with a client, measured via TF-CBT toolkit	R	C	T2-T8
TF-CBT implementation completion	Days post-training until TF-CBT initiation (screening/symptom assessment, first individual session)	R	C	T2-T5
TF-CBT fidelity	Adherence via 3 coded sessions using TF-CBT version of the Therapy Procedures Observational Coding System for Child Psychotherapy (TF-CBT TPOCS; developed by Co-I Dorsey [93]	O	O	T2-T8
TF-CBT sustainment	For clinicians who adopted TF-CBT in year 1 of their participation, re-initiation of TF-CBT in year 2	R	C	T6-T8
TF-CBT cost	Incremental costs for TF-CBT, collected via survey, records, and interview.	R, S, I	C	T5
BASIS cost	Incremental costs for BASIS, collected via survey, records, and interview.	R, S, I	C	T3
Youth clinical and functional outcomes				
Demographics	Age/grade, gender, race, ethnicity, family income, parent occupation	Q	P, Y	ST1
PTSD/PTS symptoms	Child PTSD symptoms scale for DSM-V (CPSS-V) [94]	Q	P, Y	ST1-ST3
Emotional attributions	Parent Emotional Reactivity Questionnaire (PERQ) [95]	Q	Y	ST1-ST3
Depressive symptoms	Moods and Feels Questionnaire Short Form (S-MFQ) [96]	Q	Y	ST1-ST3
Psychosocial functioning	The Strengths and Difficulties Questionnaire (SDQ) [97]	Q	P, Y	ST1-ST3
Academic outcomes	School administrative records (attendance, discipline, achievement)	R	R	ST1-ST3

Type of measure: *S* survey, *I* interview, *O* observation, *R* records. Informant: *C* clinician, *N* consultant, *O* observer, *P* parent, *R* record, *Y* youth. Timing: clinician timing—*T0* consenting, *T1-T5* baseline, *T6* time post-training, *T7* 3 months, *T8* 6 months, *T9* 9 months, *T10* sustainment 1 (fall), *T11* sustainment 2 (winter), *T12* sustainment 3 (spring); student timing—*ST1* student time 1, *ST2* student time 2, *ST3* student time 3

#### TF-CBT toolkit

Adoption and sustainment data will be collected via the TF-CBT “Toolkit,” an online TF-CBT tracking system used by clinicians to determine client eligibility, log client sessions, and facilitate consultation.

#### Qualitative interviews

To fully address aim 2 and identify factors unaccounted for by our BASIS theory of change (Fig. 1), *unexplained residuals* from aim 2 mediation analyses will be explored qualitatively via semi-structured phone interviews at the end of the active implementation phase. Residuals are defined as clinicians whose implementation behavior is insufficiently accounted for by our mediation model (e.g., clinicians with favorable implementation outcomes, but who demonstrate low levels of BASIS mechanisms). Clinicians from aim 2 will be identified at the end of their first year of participation based on the results of aim 2 quantitative modeling. Participants will include those whose predicted probability score is greater than 1.0 standard deviations of the mean from their predicted behavior, balanced between implementers and non-implementers and TF-CBT and AC conditions (approximately 15–19 clinicians total). The mixed methods design will be sequential in structure; the functions are sampling and expansion; and the process is connecting [98–100]. We will develop a systematic, comprehensive semi-structured interview guide that draws from the EPIS framework [17] to examine multilevel (i.e., intervention, individual, inner setting, outer setting) [101] determinants that explain what processes facilitated or hindered EBT implementation and sustainment.

#### Cost assessments

Activity-based costing will estimate the respective, incremental costs of enhanced TAU, TF-CBT, and BASIS. For TF-CBT/TAU costs, we will estimate direct costs of TF-CBT and enhanced TAU, such as training, consultation and delivery of services, and indirect costs, such as lost opportunities for alternative activities. For BASIS costs, we will directly measure the resource use and incremental costs associated with BASIS and TF-CBT training as compared to status quo TF-CBT training (i.e., TF-CBT implementation-as-usual) [102]. We will identify activities related to training and associated labor and non-labor inputs. Inputs can include time, supplies, travel, overhead, and costs associated with TF-CBT and BASIS training meetings, including pre-work, scheduling, etc.

#### Cost-effectiveness

We will combine incremental costs collected for BASIS, TF-CBT, and TAU with estimates of effectiveness on implementation and clinical (see “Cost and cost-effectiveness analyses” section).

### Youth data collection

Youth data collection will focus on mental health and functional outcomes targeted by TF-CBT, with evidence of effectiveness in previous studies. Youth will complete surveys via telephone prior to their first TF-CBT session (ST1) and at 3- (ST2) and 6-month (ST3) follow-ups. These surveys will assess PTSD, depression, and psychosocial functioning. On the same time frame, caregivers will complete a measure of their students’ psychosocial functioning and provide information regarding family income and parent occupation. At the end of each school year, academic records will be requested for all participants who received TF-CBT or TAU during that year. Attendance, discipline, and achievement (standardized test scores, grades) will be extracted from these academic records.

### Measures

Measures evaluate basic demographics and aspects of each school’s context, mediators (mechanisms) and moderators, implementation outcomes, and youth clinical outcomes. Table 2 displays all study measures, and Additional File 5 provides additional detail for all study measures.

### Data analytic plan

#### Aim 1: TB-CBT effects

We will test the direct main effects of TF-CBT on the hypothesized intervention mechanisms (trauma-related cognitions, emotion regulation, behavioral avoidance) and child mental health outcomes (e.g., symptoms of post-traumatic stress), via longitudinal mixed effects models (time within client within clinician) for each mechanism, using effect coding for each of the 3 conditions (BASIS, AC, enhanced TAU) in an intention-to-treat approach, and focusing our comparisons on the main effect of TAU vs. TF-CBT conditions. To increase statistical power to detect an effect of TF-CBT, we will include as covariates any variables at the client, provider, or school level that are associated with the mechanism variable, as indicated during preliminary bivariate analyses. Path analysis will then be used to test the mediated effect of mechanisms. Mixed effect models will estimate standardized coefficients for each path separately, as well as together, and any significant reduction in the condition to outcome coefficient when including the other paths will be suggestive of partial or full mediation, using the bias-corrected bootstrap method to test significance [103].

#### Aim 2: BASIS effects

For aim 2, clinicians assigned to the enhanced TAU condition will be excluded from analyses. We will test for the impact of BASIS on proximal implementation mechanisms of change via a series of piecewise longitudinal mixed effects models (time within clinician) to examine between-condition differences on the rates of change across all twelve time points pieced into three epochs (T1–T5, T6–T9, T10–T12) for each of the five primary BASIS mechanisms of change. As compared to AC, we hypothesize steeper gains for BASIS from T1 to T5, smaller rates of decline for BASIS from T6 to T9, and higher levels of sustainment for T10 to T12. The impact of BASIS on fidelity will be tested using mixed effects models, with fidelity measurement occurrence nested within clinician. Adoption and sustainment will be tested using logistic regressions using condition as a predictor (adoption: yes/no used TF-CBT in first year; sustainment: yes/no used TF-CBT in second year). We will stratify sustainment analyses (T10–T12) as follows: (1) the entire population of participants, regardless of year 1 implementation, in order to capture any sustained impact of BASIS, (2) the subgroup of clinicians who implemented in year 1, to examine the sustainment of TF-CBT implementation. Mediation will be tested as in aim 1.

Based on aim 2 quantitative models, we will identify clinicians who have a difference between predicted and actual implementation behavior of ≥ 1 SD. Transcribed data will be coded using an integrated directed content analysis [104] approach as certain codes will be conceptualized during the interview guide development (deductive approach) and other codes will be developed through a close reading of an initial subset of transcripts (inductive approach) [105]. These themes will provide a way of identifying and understanding the most salient factors that impact implementation and extend beyond the existing BASIS mechanisms and theory of change [106, 107]. Directed coding will be driven by the EPIS framework [17]. After a stable set of codes is developed, a consensus process will be used in which all reviewers independently code and compare their coding to arrive at consensus judgments through open dialog [79, 80, 108].

In all main effects analyses, the Benjamini-Hochberg procedure adjusts for familywise error within outcome families (e.g., “attitudes”). Missing data will be addressed in modeling using restricted maximum likelihood estimation.

#### Cost and cost-effectiveness analyses

For both aims, cost data collection and analyses of TF-CBT and BASIS will estimate incremental total and unit costs for TAU, BASIS+TF-CBT, and TF-CBT during both the implementation and sustainment periods [109]. We will estimate the average weighted cost metrics across the sample of study sites. For each condition, we will estimate the average incremental costs for (1) total economic costs and (2) cost per clinician trained (stratified by TAU training, BASIS training, and TF-CBT training). The cost-effectiveness analysis will compare the incremental net costs with the benefits defined as changes in clinical outcomes, such as PTS, depression, and anxiety, across the control (TAU) and the two arms TF-CBT and BASIS+TF-CBT.

### Power

#### Aim 1

Our conservative power analyses indicate that we are powered to detect minimum detectable effect sizes (MDES) of Cohen’s *d* > .35 for direct effects on student mechanisms and outcomes, assuming an intraclass correlation of .05, paired two-group (e.g., BASIS vs. enhanced TAU) tests with 35 schools/clinicians in each group (to account for attrition/missing data) and 4 clients served by each provider. Assuming some variance in the number of clients per clinician ranging from 1 to 7, a Monte Carlo simulation estimated that the maximum detrimental impact on power will be less than 10% [110]. Using bias-corrected bootstrapping, we will have sufficient power to detect mediation when each of the two mediation paths is of *d* > .2 (small effects) [111].

#### Aim 2

Conservatively assuming 35 clinicians in each group and 12 time points, we will have sufficient power to detect a Cohen’s *d* of .68 for clinician implementation outcomes, which is lower than most significant effects during our pilot study [78]. We will have power to detect mediation effects when the relationship of BASIS to implementation mechanisms is *d* > .59 (as expected) and mechanisms to implementation outcomes has at least small/moderate effects (*d* > .31) [111].

## Discussion

### Innovation

This hybrid trial will address significant gaps in implementation science surrounding the impact of pragmatic, individually focused implementation strategies and their mechanisms. It is also the first project to conduct an effectiveness trial of TF-CBT in schools. Existing compilations of implementation strategies [112, 113] contain very few individually focused strategies [114], and none are explicitly designed to impact the mechanisms identified by TPB and HAPA. In particular, although TPB is the most commonly used social-cognitive theory for designing and evaluating the impact of implementation strategies [34, 115], no studies have tested TPB constructs as mechanisms of behavior change via mediation [49, 81]. BASIS isolates individual-level mechanisms of implementation; the understanding of which will inform the design and tailoring of efficient strategies. A recent systematic review [115] identified only three prior studies that have conducted experimental tests of the impact of implementation strategies on mechanisms (mediators) and implementation outcomes. We will conduct mediation analyses testing extent to which BASIS mechanisms (TPB/HAPA constructs) account for changes in implementation outcomes as well as whether TF-CBT mechanisms account for changes in youth outcomes.

Furthermore, relative to many existing implementation strategies that unfold over months or years [116–118], the majority of BASIS activities occur at a single time point. The resources required to deliver BASIS are minimal compared to those typically invested in EBT training and consultation [119]; all of which may be wasted due to unaddressed individual-level barriers to implementation. TF-CBT has also demonstrated efficacy in improving youth symptoms, but no TF-CBT cost-effectiveness estimates [120, 121] have been conducted in schools. Cost-effectiveness is a critical but understudied factor in implementation science [122, 123] that is often a primary driver of decision-making by policymakers and leadership in service settings [124]. In the proposed trial, we will explicitly examine the cost-effectiveness of BASIS and of TF-CBT for improving youth outcomes.

Finally, much of what has been written about the sustainment phase of implementation comes from conceptual models [125, 126], and the literature has been described as “fragmented and underdeveloped” [127]. Few implementation trials have explicitly assessed the maintenance of effects produced by strategies extending into a sustainment period [128, 129]. Moreover, virtually no studies have done so in the education sector [130–132]. Given that BASIS represents a pragmatic and time-limited implementation strategy, evaluation of its long-term effects is particularly critical. Consistent with recommendations for education sector research [13], the current project will track all clinician participants into a sustainment period that spans at least one summer break and the subsequent academic year.

### Limitations

The current study only recruits one clinician per school site, which does not allow for robust evaluation of organizational covariates. We considered randomizing multiple clinicians from site to different conditions to increase sample size; however, given that BASIS is designed to target clinician perspectives of social norms, this would have presented significant risk of contamination.

## Conclusion and impact

This research will generate critical knowledge about the effectiveness and cost-effectiveness of BASIS—a pragmatic, theory-driven, and generalizable implementation strategy—to support provider behavior change, as well as the effectiveness of TF-CBT in SMH. Trial results will be disseminated via publications, presentations, via traditional (e.g., press releases) and social (e.g., Twitter) media, and through networks of practitioners. If effective, BASIS could be adapted for use in initiatives across service sectors and evaluated in subsequent trials with additional interventions. Finally, evidence supporting the effectiveness of TF-CBT in schools would be cause for increased scale-up of this EBT in the most common setting where youth with history of trauma are likely to access needed mental health services.

## Supplementary Information

**Additional file 1.** CONSORT 2010 Checklist of Information to Include When Reporting Cluster Randomised Trial.

**Additional file 2.** SPIRIT 2013 Checklist.

**Additional file 3.** IRB Approval.

**Additional file 4.** Study Consents.

**Additional file 5.** Detailed Measures Table.

## Data Availability

Please contact the lead author for more information.

## Abbreviations

AC: Attention control
AC + TF-CBT: AC plus TF-CBT
BASIS: Beliefs and Attitudes for Successful Implementation in Schools
BASIS+TF-CBT: BASIS plus TF-CBT
EBT: Evidence-based treatments
EPIS: Exploration, Preparation, Implementation, Sustainment
HAPA: Health Action Process Approach
MDES: Minimum detectable effect sizes
MI: Motivational interviewing
PTSD: Posttraumatic stress disorder
SMH: School-based mental health
TAU: Treatment-as-usual
TF-CBT: Trauma-focused cognitive behavior therapy
TPB: Theory of Planned Behavior
