# Supplementary material for: Protocol for a hybrid type 2 cluster randomized trial of trauma-focused cognitive behavioral therapy and a pragmatic individual-level implementation strategy
Source: Implement Sci. 2021 Jan 7;16:3. doi: 10.1186/s13012-020-01064-1 (PMC7788537; doi:10.1186/s13012-020-01064-1)
Supplement: Supplementary file 3 — Additional file 3. IRB Approval. [file 13012_2020_1064_MOESM3_ESM.pdf]

IRB APPROVAL OF APPLICATION

November 22, 2019

Dear Aaron Lyon:

On 11/22/2019, University of Washington IRB Committee J reviewed the following application:

Type of Review:	Initial Study
Title of Study:	A Hybrid Type 2 Trial of Trauma-Focused Cognitive Behavioral Therapy and a Pragmatic Individual-Level Implementation Strategy
Investigator:	Aaron Lyon
IRB ID:	STUDY00008401
Funding:	Name: National Institutes of Health (NIH), Grant Office ID: A143618, Funding Source ID: TBD<br>Funding Title(s): “A Hybrid Type 2 Trial of Trauma-Focused Cognitive Behavioral Therapy and a Pragmatic Individual-Level Implementation Strategy”
IND, IDE, or HDE:	None

IRB Approval

Under FWA #00006878, the IRB approved your activity.

- **Depending on the nature of your study, you may need to obtain other approvals or permissions to conduct your research. For example, you might need to apply for access to data or specimens (e.g., to obtain UW student data). Or, you might need to obtain permission from facilities managers to approach possible subjects or conduct research procedures in the facilities (e.g., Seattle School District; the Harborview Emergency Department).**
- Your application qualified for expedited review (“minimal risk”; Categories 6 & 7).
- Under the Revised Common Rule this IRB approval is valid until study completion. In other words, there is no expiration date and you are not required to submit Continuing Review Reports to maintain your approval. However, you are still required to (1) obtain IRB approval before making any changes (modifications) to your research, and (2) provide the IRB with any Reportable New Information such as breaches of confidentiality or unanticipated problems. *Note: Our current Zipline software must display an expiration date for all IRB-approved studies, so we have programmed the system to display the expiration date of 1/2/3456 for studies that will not expire.*
- This approval applies only to the activities described in your application (including any references to specific grant sections). It does not include other activities that may be described in your grant or contract.
- Your study automatically has a Certificate of Confidentiality (CoC), because you have NIH funding. A description of the CoC protections and responsibilities has been placed in your study’s Documents section.

- If you plan to continue data collection past the expiration of your NIH funding and the CoC, contact the Human Subjects Division prior to the end of your funding. We will help you determine whether you need to apply for a CoC extension.

Determinations, waivers, and regulations
The IRB made the determinations and waivers listed in the table below. Note that any granted waivers of consent or parent permission do not override a subject's refusal to provide broad consent.

Requirement	Determination or Waiver
Consent	Waived for data obtained to recruit and screen clinicians, parents and children.
Documentation of consent	Waived for all subjects; consent obtained verbally with an information sheet.
Involvement of children	Approved
Parental permission	Permission of only one parent is required
Documentation of parental permission	Waived
Assent	Required

Location of documents
Use the consent, parental permission, and assent forms that were approved and stamped by the IRB. They can be downloaded from the Final column under the **Documents tab** in Zipline.

In addition, HSD has uploaded the following documents to the **Documents tab** in Zipline:
- Certificate of Confidentiality Acknowledgement Letter

Thank you for your commitment to ethical and responsible research. We wish you great success!

Sincerely,

Lindsey Westlake
Senior Administrator
206-897-1748
scaggl@uw.edu
