# Supplementary material for: Protocol for a hybrid type 2 cluster randomized trial of trauma-focused cognitive behavioral therapy and a pragmatic individual-level implementation strategy
Source: Implement Sci. 2021 Jan 7;16:3. doi: 10.1186/s13012-020-01064-1 (PMC7788537; doi:10.1186/s13012-020-01064-1)
Supplement: Supplementary file 4 — Additional file 4. Study Consents. [file 13012_2020_1064_MOESM4_ESM.pdf]

Approved
10/2/2020
UW IRB

UNIVERSITY OF WASHINGTON
Beliefs and Attitudes for Successful Implementation in Schools (BASIS)

Youth Ages 9-12 Information Sheet

Researchers: Aaron Lyon, Ph.D., Associate Professor, Department of Psychiatry, (206) 221-8604;
Study Coordinator- Jessica Coifman (206) 543-6483

What is a research study?

Research studies help us learn new things. We can test new ideas. First, we ask a question. Then we try to find the answer.

This paper talks about our research and the choice that you have to take part in it. We want you to ask us any questions that you have. You can ask questions any time.

Important things to know…

- You get to decide if you want to take part.
- You can say 'No' or you can say 'Yes'.
- No one will be upset if you say 'No'.
- If you say 'Yes', you can always say 'No' later.
- You can say 'No' at any time.
- We would still take good care of you no matter what you decide.

Why are we doing this research?

We want to find out more about how to help kids who have had difficult experiences do their best at school. We are studying different ways to help them feel better and do better at school.

What would happen if I join this research?

If you decide to be in the research, we would ask you to do the following:

- A person on the research team would ask you some questions over the phone. Then you would say your answers out loud. We will mail you a $20 gift card for doing this.

- We would also like to ask your provider about the upsetting or stressful experience you told them about when you first met. We do this so that we can understand the kinds of things you are dealing with, and without having to make you tell us what happened to you.

- If the research team decides you are a good fit for the study, they will call you 2 more times to complete phone surveys with you and your parent/guardian — once in 3 months and again in 6 months. The overall time you would be in the study is up to 6 months. Each phone survey should take around 20 minutes. We will mail you a $20 gift card each time you complete a phone interview with us.

- Sometimes we will audio record our phone calls to make sure our staff is doing a good job. After we listen, we will destroy the recording. We will always ask you if it is ok to record the call.

- Your provider is part of the study, too. We want to know how they are doing, so we would like your

Approved
10/2/2020
UW IRB

permission to audio record (voices only) all the sessions they have with you until the study is over. We will delete the recordings but keep written notes.

- We will also ask your parent/guardian for permission to review your school records.

Could bad things happen if I join this research?

Some of the questions might make you uncomfortable or feel hard to answer. You can skip questions you do not want to answer. You can say ‘no’ to what we ask you to do for the research at any time and we will stop.

Could the research help me?

We hope this study will help kids in the future do their best at school by addressing their emotional needs.

UNIVERSITY OF WASHINGTON

Beliefs and Attitudes for Successful Implementation in Schools (BASIS)

Youth Ages 13-17 Information Sheet

Researchers: Aaron Lyon, Ph.D., Associate Professor, Department of Psychiatry, (206) 221-8604;
Study Coordinator- Jessica Coifman (206) 543-6483

RESEARCHER STATEMENT

We are asking you to be in a research study. The purpose of this form is to tell you about a research study for students and providers at your school. Since you are interested in counseling services at school, we are asking both you and your parent/guardian for their permission for you to take part in the study and also inviting your parent/guardian to participate. We will review all study information with you and you may contact us at any time if you have questions about the research.

PURPOSE OF STUDY

The purpose of this study is to learn how to help students who have had difficult experiences (trauma) improve their school performance and well-being as a result of counseling. We are studying a particular approach to addressing distressing thoughts and behaviors and how it compares to other services at school. Some schools taking part in this study will be randomly chosen to have their provider trained to provide the study intervention or to provide a resource-efficient trauma treatment. In schools where the providers receive the study training, they can use it for all students who get counseling whether or not a student is in the study. In other schools, students will receive usual school counseling. The research is funded by The National Institute of Mental Health (NIMH).

STUDY PROCEDURES

Since you are a child aged 9-17, your parent/guardian has consented and agreed that we can talk to you about this study. If you agree (also known as assent) to participate, your parent/guardian will also be invited to participate. If you do not want to participate, you do not have to even though your parent/guardian has already given permission.

We have asked your parent/guardian to complete a survey about how you have been feeling and acting to help us decide if you are eligible for the study.

If you agree to participate:

1. You will also be asked to complete a phone survey about how you have been feeling and acting to help us decide if you are eligible for the study. Whether or not you are eligible, we will mail you a $20 gift card for your time.
2. If you are eligible, we will call 2 more times to complete phone surveys with you and your parent/guardian — once in 3 months and again in 6 months. The overall duration of participation in the study will be up to 6 months. Each phone survey should take approximately 20 – 30 minutes. We will mail two gift cards for $20 each to you and your parent/guardian to thank you both for your time, each time you complete a phone interview with us.

3. To help us understand your experiences better, we will ask the provider from your school you are receiving support from for information on the stressful or upsetting events for which you are being seen. We will ask

Approved
10/2/2020
UW IRB
your provider so that you do not have to discuss your experiences beyond the support you receive from school.

4.

We may occasionally audio record our phone calls to make sure our staff is doing a good job. After we listen, we will destroy the recording. We will always ask you in advance if it is ok to record the call.

5. Your provider is part of the study, too. We want to know how they are doing, so we would like your permission to audio record (voices only) all the sessions they have with you until the study is over. The recorded sessions will be deleted but the transcripts will remain.
6. We will also ask your parent/guardian for permission to review your school records.

RISK, STRESS, OR DISCOMFORT

A standard risk is a breach of confidentiality. Some aspects of data collection will be recorded which may cause temporary discomfort to participants. You or your parent/guardian may not feel comfortable answering personal questions about feelings or behaviors. The most personal and sensitive questions we will ask you include those about your mood, feelings, and experiences. You and your parent/guardian can choose not to answer any question at any time.

You may be concerned that someone will learn about what you or your parent/guardian has said. All information shared with our research team will be private, meaning that we do not share answers with anyone outside the research team. You will not see or know what your parent/guardian says, and they will not know what you say.

ALTERNATIVES TO TAKING PART IN THE STUDY

Should you decide not take part in the study, you will still be able to meet with a provider in the schools. You can choose not to participate and receive services as usual.

BENEFITS OF THE STUDY

There are no direct benefits to you for participating in the study. We hope that this study will help young people by improving school-based mental health services. The research may lead to discoveries that will prevent problems or lead to better academic and emotional outcomes for students with emotional and behavioral problems.

CONFIDENTIALITY OF RESEARCH INFORMATION

All of the information you provide will be confidential. We take steps to guard your privacy. Your name will not be used in the research: you will be assigned a study code. We will do our very best to be sure that only the researchers see the study data. We will keep all study information in locked files that only the researchers can access. Some aspects of data collection (e.g., qualitative interviews) will be recorded and may be professionally transcribed by a company that provides secure data uploading and has adequate data protection measures in place. The data will be protected with encryption; the transfer of audio files will be done via secure, HIPAA approved cloud services. All audio files will only be listened to by members of our study staff or contracted TF-CBT fidelity coders. We will destroy all audio files and any information that could identify research participants by the end of the study. All data will be stored securely on study computers and servers without names.

The researchers will write papers and make presentations that will educate the public about what they learn from the study. No names or other identifying information will be used in any papers or presentations that may

Approved
10/2/2020
UW IRB

result from this study. When our research team looks at or reports study findings, all answers will be combined with the answers of the other participants and no names will be used. Government or university staff members sometimes review studies such as this one to make sure they are being done safely and legally. If a review of this study takes place, your data may be examined. The reviewers will protect your privacy. If you have any questions, Dr. Lyon or the research staff will answer them at any time.

If we learn that you or your parent/guardian intends to harm themselves or others, we will follow study and school policies for reporting it. By law, we must also report any suspected abuse or neglect.

OTHER INFORMATION

Participation in this project is voluntary. You may choose to participate or not or may drop out of the study at any time without any penalty or loss of benefits to which they are otherwise entitled.

<u>If your parent/guardian does not want to be interviewed at 3- and 6-months, you can still take part in this project.</u>

If you have questions later about the study or feel you or your parent/guardian has been harmed by the study, you can ask one of the researchers listed on the top of the first page. If you have questions about your rights as a research subject you can call the Human Subjects Division at the University of Washington at (206) 543-0098. Their job is to protect the rights of people who take part in research.

Approved
10/2/2020
UW IRB

UNIVERSITY OF WASHINGTON
Beliefs and Attitudes for Successful Implementation in Schools (BASIS)

Youth Ages 18-19 Information Sheet

Researchers: Aaron Lyon, Ph.D., Associate Professor, Department of Psychiatry, (206) 221-8604;
Study Coordinator- Jessica Coifman (206) 543-6483

RESEARCHER STATEMENT

We are asking you to be in a research study. The purpose of this form is to tell you about a research study for students and providers at your school. Since you are interested in counseling services at school, we are asking for your permission to take part in the study and also inviting your parent/guardian to participate. We will review all study information with you and you may contact us at any time if you have questions about the research.

PURPOSE OF STUDY

The purpose of this study is to learn how to help students who have had difficult experiences (trauma) improve their school performance and well-being as a result of counseling. We are studying a particular approach to addressing distressing thoughts and behaviors and how it compares to other services at school. Some schools taking part in this study will be randomly chosen to have their providers trained to provide the study intervention or to provide a resource-efficient trauma treatment. In schools where the providers receive the study training, they can use it for all students who get counseling whether or not a student is in the study. In other schools, students will receive usual school counseling. The research is funded by The National Institute of Mental Health (NIMH).

STUDY PROCEDURES

If you choose to participate:

1. You will complete a phone survey about how you have been feeling and acting to help us decide if you are eligible for the study. Whether or not you are eligible, we will mail you a $20 gift card for your time.

2. If you agree, we will also ask your parent/guardian to complete a survey about how you are feeling and acting to help us decide if you are eligible for the study. Whether or not you are eligible, we will mail them a $20 gift card for their time.

3. To help us understand your experiences better, we will ask the provider from your school you are receiving support from for information on the stressful or upsetting events for which you are being seen. We will ask your provider so that you do not have to discuss your experiences beyond the support you receive from school.

4. If you are eligible, we will call 2 more times to complete phone surveys with you and your parent/guardian — once in 3 months and again in 6 months. The overall duration of participation in the study will be up to 6 months. Each phone survey should take approximately 20 – 30 minutes. We will mail two gift cards for $20 each to you and your parent/guardian to thank you both for your time, each time you complete a phone interview with us.

Approved
10/2/2020
UW IRB

5. We may occasionally audio record our phone calls to make sure our staff is doing a good job. After we listen, we will destroy the recording. We will always ask you in advance if it is ok to record the call.

6. Your provider is part of the study, too. We want to know how they are doing, so we would like your permission to audio record (voices only) all the sessions they have with you until the study is over. The recorded sessions will be deleted but the transcripts will remain.

7. We will also ask you for permission to review your school records.

RISK, STRESS, OR DISCOMFORT

A standard risk is a breach of confidentiality. Some aspects of data collection will be recorded which may cause temporary discomfort to participants. You may not feel comfortable answering personal questions about feelings or behaviors. The most personal and sensitive questions we will ask you include those about your mood, feelings, and experiences. You can choose not to answer any question at any time. You may be concerned that someone will learn about what you have said. All information shared with our research team will be private, meaning that we do not share answers with anyone outside the research team. You will not see or know what your parent/guardian says, and they will not know what you say.

ALTERNATIVES TO TAKING PART IN THE STUDY

Should you decide not take part in the study, you will still be able to meet with a provider in the schools. You can choose not to participate and receive services as usual.

BENEFITS OF THE STUDY

There are no direct benefits to you for participating in the study. We hope that this study will help young people by improving school-based mental health services. The research may lead to discoveries that will prevent problems or lead to better academic and emotional outcomes for students with emotional and behavioral problems.

CONFIDENTIALITY OF RESEARCH INFORMATION

All of the information you provide will be confidential. We take steps to guard your privacy. Your name will not be used in the research: you will be assigned a study code. We will do our very best to be sure that only the researchers see the study data. We will keep all study information in locked files that only the researchers can access. Some aspects of data collection (e.g., qualitative interviews) will be recorded and may be professionally transcribed by a company that provides secure data uploading and has adequate data protection measures in place. The data will be protected with encryption; the transfer of audio files will be done via secure, HIPAA approved cloud services. All audio files will only be listened to by members of our study staff or contracted TF-CBT fidelity coders.

Consistent with NIMH policies, the information that we obtain from you for this study might be used for future studies with other authorized research teams. We may remove anything that might identify you from the information. If we do so, that information may then be used for future research studies or given to another investigator without getting additional permission from you. It is also possible that in the future we may want to use or share study information that might identify you. If we do, a review board will decide whether or not we need to get additional permission from you.

The researchers will write papers and make presentations that will educate the public about what they learn from the study. No names or other identifying information will be used in any papers or presentations that may

Approved
10/2/2020
UW IRB

result from this study. When our research team looks at or reports study findings, all answers will be combined with the answers of the other participants and no names will be used. Government or university staff members sometimes review studies such as this one to make sure they are being done safely and legally. If a review of this study takes place, your data may be examined. The reviewers will protect your privacy. If you have any questions, Dr. Lyon or the research staff will answer them at any time. If we learn that you or your parent/guardian intends to harm themselves or others, we will follow study and school policies for reporting it.

We have a Certificate of Confidentiality from the National Institutes of Health. This helps us protect your privacy. The Certificate means that we do not have to give out identifying information about you even if we are asked to by a court of law. We will use the Certificate to resist any demands for identifying information.

We can't use the Certificate to withhold your research information if you give your written consent to give it to an insurer, employer, or other person. Also, you or a member of your family can share information about yourself or your part in this research if you wish.

There are some limits to this protection. We will voluntarily provide the information to:

- a member of the federal government who needs it in order to audit or evaluate the research;
- individuals at the University of Washington, the funding agency, and other groups involved in the research, if they need the information to make sure the research is being done correctly;
- the federal Food and Drug Administration (FDA), if required by the FDA;
- Local or state authorities, if we learn of child abuse, elder abuse, or the intent to harm yourself or others.

OTHER INFORMATION

Participation in this project is voluntary. You may choose to participate or not or may drop out of the study at any time without any penalty or loss of benefits to which they are otherwise entitled.

A description of this clinical trial will be available on http://www.clinicaltrials.gov, as required by U.S. Law. This Web site will not include information that can identify you. At most, the Web site will include a summary of the results. You can search this Web site at any time.

If you have questions later about the study or feel you have been harmed by the study, you can ask one of the researchers listed on the top of the first page. If you have questions about your rights as a research subject you can call the Human Subjects Division at the University of Washington at (206) 543-0098. Their job is to protect the rights of people who take part in research.

Approved
8/17/2020
UW IRB

UNIVERSITY OF WASHINGTON
SMART Center Trauma-Informed Intervention Project

Provider Information Sheet

Researchers:
Aaron Lyon, Ph.D., Associate Professor, Department of Psychiatry, (206) 221-8604;
Chayna Davis, Ph.D., Research Lead, Department of Psychiatry, (206) 221-9087

RESEARCHER STATEMENT

We are asking you to be in a research study. The purpose of this form is to help you decide whether to be in the study or not. Should you have any questions about the purpose of the research, what we are asking you to do, the possible risks and benefits, your rights as a volunteer, or anything else about the research or this form, please contact the research lead. If you are willing to be part of this study, we are asking you to consent using this web-based form.

PURPOSE OF STUDY

The goals of the study are to (1) evaluate the effectiveness of two evidence-informed interventions for youth trauma with students and to (2) evaluate the impact of two strategies for enhancing intervention adoption and use. The research is funded by The National Institute of Mental Health (NIMH).

STUDY PROCEDURES

You are one of approximately 120 School Mental Health providers being invited to take part in the study. Your participation will last 18-21 months.

If you agree to take part, we will ask you to:

- Complete web-based surveys about your perceptions of evidence-based treatments up to 12 times over two years. Each survey will take approximately 5 to 15 minutes.
- Agree to be randomized to either a Trauma Focused Cognitive Behavioral Therapy (TF-CBT) condition (and one of the two strategies for enhancing adoption and use) or a Resource Efficient Trauma Intervention (RETI) condition.
- Assess students referred to you for appropriateness for TF-CBT or RETI treatment and the study using a measure we will provide.
- Deliver TF-CBT or RETI treatment to 4-6 students enrolled in the study.
- Once the researchers enroll a student into the study, record your treatment sessions with the student (students and parents will give permission for the recording during the research enrollment process).
- Possible participation in a 30-45 minute qualitative interview if selected randomly (up to 20 providers only).

If you are randomized to TF-CBT, we will ask you to:

- Complete training and become certified in TF-CBT. This involves:
  - Complete an online TF-CBT training. You will receive a $100 gift card upon completion;
  - Attend a multi-day virtual or in-person training;
  - Participate in 6 months (twice a month) of remote consultation.

If you are randomized to RETI, we will ask you to:

- 4 – 6 hours of RETI training.

Approved
8/17/2020
UW IRB

RISK, STRESS, OR DISCOMFORT

A standard risk is a breach of confidentiality. Some aspects of data collection will be recorded which may cause temporary discomfort to providers.

BENEFITS OF THE STUDY

There are no direct benefits to you for participating in the study. You may appreciate the opportunity to contribute to the larger body of knowledge related to improving services for students in schools. The research may lead to discoveries that enhance the understanding of how to successfully implement evidence-based practices in schools that will prevent problems or lead to better academic and emotional outcomes for students.

CONFIDENTIALITY OF RESEARCH INFORMATION

All of the information you provide will be confidential. We take steps to guard your privacy. Your name will not be used in the research: you will be assigned a study code. We will do our very best to be sure that only the researchers see the study data. We will keep all study information in locked files that only the researchers can access. Some aspects of data collection (e.g., qualitative interviews) will be recorded and may be professionally transcribed by a company that provides secure data uploading and has adequate data protection measures in place. The data will be protected with encryption; the transfer of audio files will be done via secure, HIPAA approved cloud services. All audio files will only be listened to by members of our study staff or contracted TF-CBT fidelity coders.

Consistent with NIMH policies, the information that we obtain from you for this study might be used for future studies with other authorized research teams. We may remove anything that might identify you from the information. If we do so, that information may then be used for future research studies or given to another investigator without getting additional permission from you. It is also possible that in the future we may want to use or share study information that might identify you. If we do, a review board will decide whether or not we need to get additional permission from you.

The researchers will write papers and make presentations that will educate the public about what they learn from the study. No names or other identifying information will be used in any papers or presentations that may result from this study. When our research team looks at or reports study findings, all answers will be combined with the answers of the other participants and no names will be used. Government or university staff members sometimes review studies such as this one to make sure they are being done safely and legally. If a review of this study takes place, your data may be examined. The reviewers will protect your privacy. If you have any questions, Dr. Lyon or the research staff will answer them at any time.

We have a Certificate of Confidentiality from the National Institutes of Health. This helps us protect your privacy. The Certificate means that we do not have to give out identifying information about you even if we are asked to by a court of law. We will use the Certificate to resist any demands for identifying information.

We can't use the Certificate to withhold your research information if you give your written consent to give it to an insurer, employer, or other person. Also, you or a member of your family can share information about yourself or your part in this research if you wish.

There are some limits to this protection. We will voluntarily provide the information to:

- a member of the federal government who needs it in order to audit or evaluate the research;
- individuals at the University of Washington, the funding agency, and other groups involved in the research, if they need the information to make sure the research is being done correctly;
- the federal Food and Drug Administration (FDA), if required by the FDA;

Approved
8/17/2020
UW IRB

- Local or state authorities, if we learn of child abuse, elder abuse, or the intent to harm yourself or others.

OTHER INFORMATION

Participation in this project is voluntary. You may choose not to participate or may withdraw from the study at any time without any penalty or loss of benefits to which they are otherwise entitled. Study results will be provided to participating organizations.

If you choose to participate, we will send you a $400 gift card as a thank you for your participation in the active treatment phase (year 1) for completing web-based surveys and delivering treatment to students. We will later send you a $200 gift card as a thank you for your participation in the sustainment phase (year 2) for completing web-based surveys after treatment with all students is completed.

A description of this clinical trial will be available on http://www.clinicaltrials.gov, as required by U.S. Law. This Web site will not include information that can identify you. At most, the Web site will include a summary of the results. You can search this Web site at any time.

If you have questions about the research or if you have been harmed by the study, please contact one of the researchers listed on the top of the first page. Please indicate your willingness to take part in this study or not by checking the box below. Checking the box “YES to study participation” will result in linking you to the study survey. If you have questions about your rights as a research subject, you can call the Human Subjects Division at (206) 543-0098.

❑ YES to participation.

❑ NO to participation.
