# Supplementary material for: Protocol for a hybrid type 2 cluster randomized trial of trauma-focused cognitive behavioral therapy and a pragmatic individual-level implementation strategy
Source: Implement Sci. 2021 Jan 7;16:3. doi: 10.1186/s13012-020-01064-1 (PMC7788537; doi:10.1186/s13012-020-01064-1)
Supplement: Supplementary file 5 — Additional file 5. Detailed Measures Table. [file 13012_2020_1064_MOESM5_ESM.docx]

| **Name / Construct** | **Type (i.e., primary, secondary, other)** | **Time Frame** | **Description / Instrument** |
| --- | --- | --- | --- |
| Attitudes | Primary | Pre-Training (T1), Post Training (T2-T5), 1 month (T6),3 month (T7), 6 month (T8), 9 month (T9), 12 month (T10), 15 month, (T11), 18 month (T12) | The school-adapted version of the Evidence-Based Attitudes Scale (EBPAS)[119,132] is a widely-used 15-item tool designed to assess EBP attitudes with 4 subscales. Items are rated on a five-point scale (0 = “Not at All” to 4 = “To a Very Great Extent”). Internal reliabilities are adequate for the total score (alpha = .81) and the subscale scores: (1) appeal of EBPs (alpha = .77), (2) openness to new practices (alpha = .84), (3) likelihood of adopting an EBP (alpha = .60), and (4) perceived divergence between current practices and EBPs (alpha = .67). Construct validity has been demonstrated via factor structure replication in cross-cultural samples. |
| Subjective social norms | Primary | Pre-Training (T1), Post Training (T2-T5), 1 month (T6),3 month (T7), 6 month (T8), 9 month (T9), 12 month (T10), 15 month, (T11), 18 month (T12) | The modified Subjective Norms measure, used in previous studies and based on guidelines for developing reliable and valid measures of Theory of Planned Behavior (TPB) constructs,[120,132] includes 8 items examining injunctive and descriptive EBP implementation norms. Subjective norm scales have demonstrated acceptable reliability (alpha >.74). In our studies, we found construct validity for this measure as it was positively associated with a social norms influencing intervention. |
| Self-efficacy and Maintenance Self-efficacy | Primary | Pre-Training (T1), Post Training (T2-T5), 1 month (T6),3 month (T7), 6 month (T8), 9 month (T9), 12 month (T10), 15 month, (T11), 18 month (T12) | A modified version of the Teacher Self-Efficacy Scale [86] has 10 items assessing perceived behavioral control in implementing EBT. Item wording has been modified to be consistent with self-efficacy as it relates to the implementation of EBTs. This scale assesses teachers’ confidence and self-efficacy regarding teaching practices. The measure has demonstrated sufficient internal consistency (alpha = .82), test-retest reliability (r = .65 for over two year), and criterion validity. The modified Teacher Self Efficacy Scale will be used to evaluate both initial self-efficacy and, over time, maintenance self-efficacy. |
| Implementation intentions | Primary | Pre-Training (T1), Post Training (T2-T5), 1 month (T6),3 month (T7), 6 month (T8), 9 month (T9), 12 month (T10), 15 month, (T11), 18 month (T12) | The Modified Intentions to Use Scale [122] includes 5 items, modified to specifically assess SBMH providers’ intentions to use EBT. This scale was developed based on established guidelines for developing behavioral intention measures using the TPB. [120,132] |
| Trauma-related cognitions | Primary | Pre-Treatment (ST1); Post-treatment (ST2); 6-month follow-up (ST3) | The Child Post-Traumatic Cognitions Inventory (CPTCI) [123] is a 25-item measure for youth aged 6-18 that is applicable to a wide range of trauma experiences. Two subscales, (1) Permanent and disturbing change (in response to trauma) and (2) Fragile person in a scary world have been identified. Both subscales have high internal consistency (alpha = .91 and .87, respectively) and test-retest reliability (r = .78 and .72). |
| TF-CBT consultation participation | Primary | Post-training (T5), 3 month (T7), 6 month (T8) | Ongoing participation will be operationalized by a number of indicators, including (1) attending at least one post-training consultation session, (2) number (%) of consultation sessions attended, (3) days post-training to consultation dropout, defined by verbal/written withdrawal from consultation, or 3 consecutive missed consultation sessions, |
| TF-CBT adoption | Primary | Pre-Training (T1), Post Training (T2-T5), 1 month (T6),3 month (T7), 6 month (T8), 9 month (T9), 12 month (T10), 15 month, (T11), 18 month (T12) | Adoption is operationalized as the initiation of a clinician’s first TF-CBT session at any point during study participation. These data will be collected from an online TF-CBT “Toolkit” that facilitates tracking of services delivered and has been commonly used by Co-I Dorsey for large-scale TF-CBT implementation. |
| TF-CBT implementation completion | Primary | Pre-Training (T1), Post Training (T2-T5), 1 month (T6),3 month (T7), 6 month (T8), 9 month (T9), 12 month (T10), 15 month, (T11), 18 month (T12) | Implementation completion will be operationalized as (1) Number of days between TF-CBT training and initiation of clinicians’ caseload trauma screening; (2) Number of days between TF-CBT training and first TF-CBT session; (3) Number of days between TF-CBT training and completion of first full TF-CBT case. |
| TF-CBT fidelity | Primary | Following first group initiation (Post-training [T6] – 6 month [T7]); Fall of Year 2 (9 month [T8] – 12 month [T12]) | For each case, one recording will be randomly selected from each TF-CBT treatment phase and coded for fidelity using the established TF-CBT version of the Therapy Process Observational Coding System for Child Psychotherapy (TF-CBT TPOCS; developed by Co-I Dorsey).[128] |
| TF-CBT sustainment | Primary | 12 month (T10), 15 month (T11), 18 month (T12) | For clinicians who adopted TF-CBT in Year 1 of their participation, re-initiation of TF-CBT with their caseload in Year 2. |
| Costs (TF-CBT and BASIS) | Secondary | Post-Training (T5) | We will identify activities related to training, and associated labor and non-labor inputs. Inputs can include time, supplies, travel, overhead, and costs associated with TF-CBT and BASIS training meetings, including pre-work, scheduling, and attending meetings. For TF-CBT, this will include initial training, consultations, intervention delivery, etc. For BASIS, this will include the initial session, the post-training session, and the booster. For both trainings, the prices of goods and services will be available from project expense reports, which we will use to capture all costs associated with planning, materials development, training, and procurement. To capture clinician time, clinicians will complete questionnaires assessing time and resources use immediately post-training and throughout the consultation period. |
| Emotional Regulation | Primary | Pre-Treatment (ST1); Post-treatment (ST2); 6-month follow-up (ST3) | The Emotional Regulation Questionnaire (ERQ) [124] is a 10-item self-report scale designed to assess habitual use of two commonly used strategies to alter emotion: cognitive reappraisal and expressive suppression. Participants respond to each item using a 7-point Likert scale ranging from 1 (strongly disagree) to 7 (strongly agree). |
| Behavioral Avoidance | Primary | Pre-Treatment (ST1); Post-treatment (ST2); 6-month follow-up (ST3) | The Posttraumatic Avoidance Behavior Questionnaire (PABQ) [125] is a 25-item inventory, consisting of seven subscales measuring typical situations and activities that PTSD patients avoid. |
| PTSD symptoms | Primary | Pre-Treatment (ST1); Post-treatment (ST2); 6-month follow-up (ST3) | The Child PTSD Symptoms scale for DSM-V (CPSS-V) [129] is the 20 PTSD symptom items are rated on a 5-point scale of frequency and severity from 0 (not at all) to 4 (6 or more times a week /severe). The 7 functioning items are rated on yes/no. |
| Emotional Attributions | Primary | Pre-Treatment (ST1); Post-treatment (ST2); 6-month follow-up (ST3) | The Parent Emotional Reactivity Questionnaire (PERQ) [130] is designed to assess stressful parental emotional reactions to the sexual abuse of their children. Parents are asked to endorse the frequency of specific reactions including fear, sadness, guilt, anger, embarrassment, shame, and emotional preoccupation. The measure yields a total PERQ score. |
| Depressive symptoms | Primary | Pre-Treatment (ST1); Post-treatment (ST2); 6-month follow-up (ST3) | The Moods and Feelings Questionnaire Short Form (S-MFQ) [131] consists of a series of 13 descriptive phrases regarding how the subject has been feeling or acting recently. It is a screening tool for depression in children and young people aged 6 to 19. |
| Psychosocial functioning | Primary | Pre-Treatment (ST1); Post-treatment (ST2); 6-month follow-up (ST3) | The Strengths and Difficulties Questionnaire (SDQ) [132] is 25 items and address 5 domains (emotional distress, conduct problems, attention, peer relationships, prosocial behavior). The SDQ demonstrates good psychometrics cross-culturally, and is sensitive to change. |
| Academic outcomes | Secondary | Pre-Treatment (ST1); Post-treatment (ST2); 6-month follow-up (ST3) | School administrative records maintained by participating districts will be supplied to the research team for participating students (see Letters of Support). Administrative data will include: (1) School attendance (truancies, absences, and daily attendance), (2) disciplinary actions (office discipline referral, in- and out-of-school suspension and expulsion), and (3) academic achievement (i.e., grades, standardized test scores, credits earned). |
| Clinician demographics | Other | Baseline (T0) | Age, gender, race/ethnicity, education level, years of experience on the job, current case, work activities performed, and prior experience with EBT. |
| Parent Demographics | Other | Pre-Treatment (ST1) | Parent relationship to child, birthday, age, gender, race/ethnicity, education, primary language, secondary language, and income. |
| Child Demographics | Other | Pre-Treatment (ST1) | Age, gender, race/ethnicity, birthdate, primary languages, and secondary languages. |
| School characteristics | Other | Baseline (T0) | School size, % eligible for free/reduced lunch, racial/ethnic composition, % English Language Learners, % in special education, annual funding for external resources, per capita number of community-based organizations providing services in the school. |
| Implementation climate | Other | Baseline (T0); 12 month (T6) | The school-adapted Implementation Climate Assessment (ICA) [126,135] includes 18 items with 6 subscales. Focus on EBT, Educational Support for EBT, Recognition for EBT, Rewards for EBT, Selection for EBT, and Selection for Openness, with subscale internal consistency ranging from .81 to .91. The ICA correlates moderately with, but is distinct from, a conceptually similar strategic climate measure. |
| Implementation leadership | Other | Baseline (T0); 12 month (T6) | The school-adapted Implementation Leadership Scale (ILS) [127,135] includes 12 items with 4 subscales: Proactive Leadership (anticipating and addressing implementation challenges), Knowledgeable Leadership (deep understanding of EBP and implementation issues), Supportive Leadership (support for EBP adoption/use), and Perseverant Leadership (consistent and responsive to challenges). Subscale internal consistencies range from 0.95 to 0.98. |
| Implementation determinants | Other | Between Post-Training (T5) and Month 1 (T6) | Drawing from the EPIS framework, a semi-structured phone interview (30-45 minutes) developed to investigate factors that explain why TPB mechanisms may be connected to outcomes in ways unaddressed by our BASIS theory of change. The interview will examine multilevel (i.e., intervention, individual, inner setting, outer setting) determinants to explain what processes facilitated or hindered EBP implementation. |
| BASIS (implementation strategy) fidelity | Other | Post-training (T5) | Core components of BASIS will be rated on two dimensions of fidelity (adherence and participant responsiveness) using the BASIS implementation strategy fidelity instrument (BASIS-ISFI), which was developed during the original BASIS R21.[79] Each videotape will be rated by two trained coders using a consensus process. |
